# Tumor Dormancy: Implications for Invasion and Metastasis

**DOI:** 10.3390/ijms22094862

**Published:** 2021-05-04

**Authors:** Georgia Gomatou, Nikolaos Syrigos, Ioannis A. Vathiotis, Elias A. Kotteas

**Affiliations:** Oncology Unit, Third Department of Medicine, Sotiria General Hospital, National and Kapodistrian University of Athens, 11527 Athens, Greece; georgiagom@med.uoa.gr (G.G.); nksyrigos@gmail.com (N.S.); ioannis.vathiotis@yale.edu (I.A.V.)

**Keywords:** tumor dormancy, cellular dormancy, tumor recurrence, metastasis

## Abstract

Tumor dormancy refers to a critical stage of cancer development when tumor cells are present, but cancer does not progress. It includes both the concept of cellular dormancy, indicating the reversible switch of a cancer cell to a quiescent state, and that of tumor mass dormancy, indicating the presence of neoplastic masses that have reached cell population equilibrium via balanced growth/apoptosis rates. Tumor dormancy provides the conceptual framework, potentially explaining a major challenge in clinical oncology, tumor recurrence, which may occur years after cancer diagnosis. The mechanisms by which tumors are kept dormant, and what triggers their reawakening, are fundamental questions in cancer biology. It seems that a plethora of intracellular pathways and extracellular factors are involved in this process, rewiring the cells to plastically alter their metabolic and proliferative status. This phenomenon is highly dynamic in space and time. Mechanistic insights into both cellular and tumor dormancy have provided the rationale for targeting this otherwise stable period of cancer development, in order to prevent recurrence and maximize therapeutic benefit.

## 1. Introduction

Tumor recurrence, which may occur even many years after curative therapeutic approaches, represents a major problem in clinical oncology [1]. Although adjuvant therapy, administered in certain cases, is considered to prevent relapse by targeting residual disease, not all patients benefit from it. Additionally, it has been observed that the tumor latency period differs substantially among different neoplasms; in fact, certain cancers, such as hormone receptor-positive breast cancer, are typically characterized by prolonged metastatic potential [2,3,4].

Tumor dormancy marks a critical phase in cancer development in which tumor cells are present, but tumor progression is not clinically apparent [5]. This term includes both cellular, referring to a reversible, non-proliferative, but viable cell status, and tumor mass dormancy, referring to the existence of neoplastic masses that do not progress [5]. During the last decades, substantial progress has been made towards deciphering the mechanisms underlying tumor dormancy [6,7]. Moreover, tumor dormancy is not only an intriguing conceptual framework theoretically explaining cancer recurrence and metastasis, but also a clinically relevant phenomenon with potential implications in cancer diagnostics and therapeutics [7]. To this end, this review article aims to provide mechanistic insight into tumor dormancy and discuss its implications in tumor invasion and metastasis.

## 2. Cellular and Tumor Dormancy: Terminology

The current paradigm of dormancy includes two potential scenarios; either the cell cycle of each cancer cell reversibly ceases (cellular dormancy) or the growth/apoptosis rate of the entire tumor mass achieves an equilibrium, potentially induced by extracellular mechanisms (tumor mass dormancy) [8,9]. In the first case, the term cellular dormancy is, in practice, used interchangeably with the term quiescence, representing a reversible, non-proliferative, but viable cell status. Of note, it should be differentiated from cellular senescence, which, contrary to quiescence, is irreversible [10,11]. In the second case, the term tumor dormancy refers to masses that include at least a part of proliferating cells [9]. A solid body of evidence has demonstrated that two main extracellular factors may affect the cell population equilibrium; (a) hypoxia, and (b) the immune system [9].

Given the above, it has been suggested that tumor dormancy could be divided into three categories, which are not necessarily mutually exclusive [12]: (a) cellular dormancy, where solitary or small groups of cells enter quiescence driven by intrinsic and/or extrinsic mechanisms; (b) angiogenic dormancy, where poor vascularization of the tumor mass leads to a balance between dividing and apoptotic cells; (c) immune-mediated dormancy, where a proliferating tumor mass is kept constant due to the cytotoxic effect of the immune system. The above processes could be complementary, as in the scenario of quiescent cells that evade immune surveillance, but as they later switch into proliferation, they may be maintained in equilibrium by immune cytotoxicity [9,12].

Interestingly, there are similarities between the concept of tumor dormancy and the cancer stem cell (CSC) theory of tumor development [13]. The CSC hypothesis includes a subgroup of cancer cells with self-renewal capacity that fuels tumor initiation, potentially survives anticancer treatment, and may cause cancer recurrence and metastatic progression [8,14,15]. Crea et al. suggested an integrated theory of cellular dormancy and CSC theory. Cancer cells could be broadly classified into dormancy-competent CSCs, cancer-repopulating cells, dormancy-incompetent CSCs, and disseminated tumor cells. Dormancy-competent CSCs are the subset of cancer stem cells that can plastically switch between dormancy and proliferation [16]. The authors propose that dormancy-competent cancer stem cells may be present at the initial phases of tumor development but as genetic alterations accumulate, they progressively lose their dormant potential so that more advanced tumors mostly contain highly mutated dormancy-incompetent CSCs, which fuel uncontrolled proliferation [16,17].

## 3. The Role of Tumor Dormancy in the Invasion-Metastasis Cascade

The majority of cancer-associated deaths are caused by metastatic disease rather than primary tumor [18]. Metastasis formation is a multistep process involving local invasion from the primary tumor, intravasation, survival in circulation, extravasation, and survival and proliferation in a target organ [19]. The above process, referred to as the invasion-metastatis cascade, is a rather inefficient procedure since only a small proportion of cancer cells, termed disseminating tumor cells (DTCs), reach the final step of distant organ colonization [9]. Initially, a fraction of cancer cells acquire traits that enable them to invade and metastasize; notably via epithelial–mesenchymal transition (EMT), a biological process involving loss of epithelial and gain of mesenchymal characteristics [19]. Cancer cells might invade surrounding tissues, mostly in groups of cells, the leading front of which has the potential to release proteases and invade [19]. Upon intravasation into the bloodstream, the cells, now termed circulating tumor cells (CTCs) encounter the environmental hazards posed by the circulatory and the immune system, however, a fraction of them either individually or in clusters, surpass the risk, for instance by interacting with platelets [20]. Eventually, the adhesion of CTCs to endothelial cells is the first step of the extravasation. Subsequent to trans-endothelial migration, cancer cells will either enter a dormant or a proliferative state [20,21].

In fact, there are at least three circumstances in which cancer cells may switch to a dormant phenotype in order to adapt and survive: (a) primary cancer dormancy; (b) metastatic dormancy; and (c) therapy-induced dormancy [22,23]. In primary tumors, cancer cells alternate between proliferation and quiescence in order to gain the molecular characteristics that are necessary for survival [16,24]. Metastatic dormancy refers to DTCs that become dormant while adapting to the microenvironment of the bloodstream or the target organ [9]. Finally, therapy-induced dormancy describes cancer cells that enter a dormant phase to escape from the effects of anticancer therapy, which usually targets proliferating cells [7].

From a clinical perspective, tumor dormancy, and metastatic dormancy, in particular, could be monitored as an indicator of tumor recurrence during patients’ follow-up. In the case of breast cancer, for example, the presence of DTCs in the bone marrow at diagnosis or after (neo)adjuvant therapy has been correlated with poor prognosis [25,26,27,28]. DTC status has been successfully used to identify high-risk patients after adjuvant chemotherapy in order to receive secondary treatment with docetaxel [29]. However, apart from enumeration, it is important to note that the functional status of DTCs should be assessed as well [30]. To this end, characterization of the dormancy status of DTCs would be of utmost importance; however, very few studies exist in this direction [9]. In a recent study, nuclear receptor subfamily 2, group F, member 1 (NR2F1), a dormancy marker identified in experimental studies, was used to stratify DTCs. Patients with NR2F1-high DTCs had longer bone metastasis-free periods than those with NR2F1-low DTCs [30]. As the understanding of tumor dormancy and the identification of its mechanisms is increasing, close collaboration of basic scientists with clinicians is essential in order to fuel clinical translation, which is still in early steps. In the following sections, we provide an overview of the mechanisms implicated in tumor dormancy phenomenon and suggested therapeutic approaches.

## 4. Mechanisms of Dormancy Induction and Maintenance

Evolutionarily, it has been suggested that cellular dormancy is a conserved mechanism that helps cells or organisms adapt to stress stimuli and survive a hostile environment [12,31,32]. Examples of environmental adaptation include the case of *Caenorhabditis elegans*, where nutrient deprivation activates stress pathways leading to cellular dormancy and growth arrest [33], and that of memory T cells of mammalian organisms, that, in the absence of an antigen, enter a dormant status associated with low energy utilization and proliferation, to survive until they receive stimulatory signals during another infection [34].

Abundant evidence from basic research and clinical studies demonstrate that the induction of dormancy and the maintenance of the dormant phenotype is a complex process [5,6], mediated by intrinsic and autocrine signaling, as well as signals derived from immune cells, endothelial cells, and other components of the tumor microenvironment [5].

### 4.1. Intracellular Mechanisms

#### 4.1.1. Genetic Alterations

Several genetic alterations, mostly associated with cell proliferation and/or differentiation, have been correlated with the initiation and maintenance of the dormant phenotype in various preclinical cancer models. In a breast cancer model, upregulation of FBXW7 gene, which encodes a component of an Skp1–Cul1–F box–type (SCF-type) E3 ubiquitin ligase leading to degradation of the positive cell-cycle regulators cyclin E and c-Myc, was associated with the dormancy of disseminated tumor cells (DTCs) [35]. Genetic ablation of FBXW7 drove DTCs to exit quiescence and start dividing, in both mouse xenograft and allograft models [35]. Another study demonstrated that leukemia inhibitory factor receptor (LIFR), whose ligand is a member of the interleukin-6 (IL-6) family of cytokines, confers a dormant phenotype of disseminated breast tumor cells [36]. Loss of the LIFR or its downstream target signal transducer and activator 3 (STAT3) was linked to downregulation of dormancy-associated genes and entrance to proliferation [37]. In a model of head and neck squamous cell carcinoma (HNSCC), paired-related homeobox transcription factor (PRRX1), which functions as a transcription coactivator and has already been implicated in the epithelial-to-mesenchymal transition program, was shown to reduce the levels of miR-642b-3p, which mediated cell dormancy of HNSCC cells through TGF-β2 and p38. The authors raised the possibility that EMT may help keep cancer cells in the dormant state [38]. Upregulation of the KiSS1 gene, which encodes the protein Kisspeptin, has been associated with inhibition of metastasis and maintenance of dormancy in preclinical models of breast cancer, ovarian cancer, and melanoma [39,40,41].

#### 4.1.2. Autophagy-Related Alterations

It has been suggested that autophagy is a pivotal mechanism for the survival of dormant DTCs. Autophagy is a mechanism of cell survival, which involves the degradation of organelles, misfolded proteins, and parts of the cytosol, to recycle dysfunctional macromolecules, and provide appropriate energy balance under metabolic stress conditions [42]. Dormant cancer cells have been shown to be more autophagic compared to proliferating cells [43]. It is presumed that dormant cells activate autophagy in order to survive oxidative stress, with improved cellular bioenergetics [42]. In a recent study, pharmacologic or genetic inhibition of autophagy-related 7 gene (ATG7) in dormant breast cancer cells, resulted in significantly decreased cell survival [43]. Moreover, re-expression of the tumor suppressor gene aplasia Ras homolog member I (ARHI; also known as DIRAS3), which is maternally imprinted, and is frequently downregulated in ovarian cancer, promoted autophagy through inhibition of phosphatidylinositol 3-kinase (PI3K) signaling and mammalian target of rapamycin (mTOR), and upregulation of ATG4 cysteine protease. Interestingly, although ARHI re-expression led to autophagic cell death when ovarian cancer cells were grown in culture, it enabled the cells to remain dormant when they were grown in mice as xenografts [44].

#### 4.1.3. Intracellular Signaling

Importantly, the balance between activated extracellular signal-regulated kinases (ERK1/2) and activated p38α/β was the first signaling mechanism that has been correlated reproducibly to cell dormancy in vitro and in vivo in multiple preclinical models [45,46,47,48,49]. In fact, it has been suggested that the ERK/p38 ratio is indicative of the dormant phenotype; a high ratio induces tumor growth, whereas a low ratio promotes tumor dormancy [48]. The protein p38 is a stress-activated protein kinase that drives a downstream program that resembles endoplasmic reticulum (ER) stress and coordinates a stress-related transcriptional program [50]. Specifically, there is evidence that the activation of p38 MAPK in dormant cells induces the unfolded-protein response (UPR), a central cellular response under stress conditions [50]. Regarding other signaling pathways, in a recent study of in vitro and in vivo models of estrogen receptor-positive (ER+) breast cancer, it was reported that the canonical activation of the NFκB pathway promoted a dormant, metastatic phenotype in ER+ breast cancer [51]. Finally, reduced PI3K/AKT signaling, mainly due to extracellular regulation as a response to environmental stimuli, has been shown to play a role in switching between cell proliferation and dormancy [52,53].

#### 4.1.4. Epigenetic Mechanisms

The ability of dormant residual tumor cells to alternate between dormancy and proliferation may be the result of epigenetic reprogramming mechanisms, namely DNA methylation, histone modifications, and non-coding RNAs [54,55]. The orphan nuclear receptor NR2F1 is epigenetically downregulated via promoter hypermethylation in various cancers but becomes highly expressed in models of dormancy [56]. NR2F1-induced quiescence is mediated by transcription factor SOX9, retinoic acid receptor β (RARβ), and cyclin-dependent kinase (CDK) inhibitors. Additionally, NR2F1 induces global chromatin repression by activating the pluripotency gene NANOG, which contributes to the dormancy of disseminated tumor cells in the bone marrow. Moreover, NR2F1 as well as RARβ direct the deacetylation of histone H3 by histone deacetylases, which is associated with the presence of dormant disseminated tumor cells in vivo [56]. Additionally, it has been shown that a downstream target of p38, the mitogen- and stress-activated protein kinase 1 (MSK1) controls markers of stemness and differentiation via phosphorylation of histone H3 at serine 10 or serine 28 [57]. Concerning deregulation of microRNAs, Almong et al. showed that micro-tumor passage from dormant to a proliferating phenotype is governed by a stable miRNA switch and reported the identification of a consensus signature of human tumor dormancy-associated miRNAs (DmiRs) in human dormant breast carcinoma, glioblastoma, osteosarcoma, and liposarcoma tumors. Restoration of a single DmiR (miR-580, 588, or 190) led to a phenotypic reversal of fast-growing angiogenic tumors towards prolonged tumor dormancy [58]. Further research specifically on miR-190 demonstrated that its upregulation was associated with prolonged tumor dormancy effects and that it affects several transcriptional factors, tumor suppressor genes, and interferon response pathways [58,59]. In another study, upregulation of miR-101 concomitantly activated several pathways including EZH2- and TP53-related proteins associated with dormant cancer stem cell phenotype [60].

**Figure 1:**
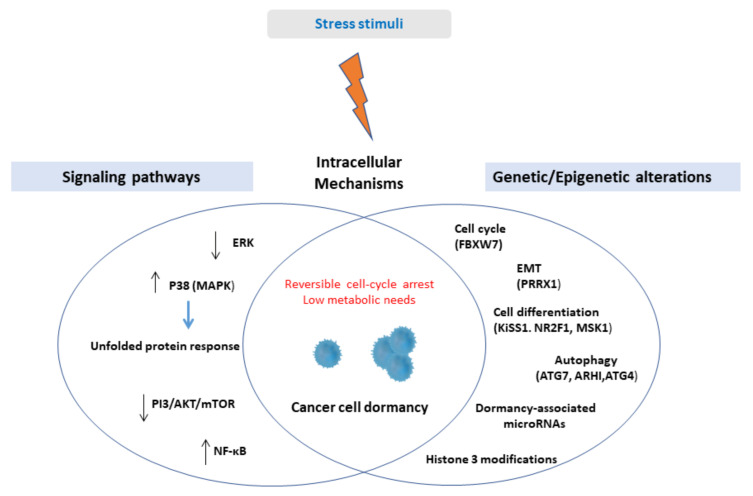
Upon stress stimuli, solitary cancer cells or small clusters of cells might activate cell-intrinsic mechanisms in order to enter a reversible state of non-proliferation and low bioenergetics. Cell-intrinsic mechanisms include genetic and epigenetic alterations associated with crucial cell functions, namely cell proliferation, cell differentiation, epithelial-mesenchymal transition, autophagy, and deregulation of several signaling pathways, among them, the most studied being the upregulation of p38 kinase. Abbreviations: ERK = extracellular signal-regulated kinase, MAPK = mitogen-activated protein kinase, PI3 = phosphatidylinositol 3-kinases, AKT = protein kinase B, mTOR = mammalian target of rapamycin, NF-κΒ = nuclear factor kappa light chain enhancer of activated B cells, EMT = epithelial-mesenchymal transition, FBXW7 = F-box and WD repeat domain containing 7, PRRX1 = paired-related homeobox transcription factor, NR2F1 = nuclear receptor subfamily 2, group F, member 1, MSK1 = mitogen- and stress-activated protein kinase 1, ATG7 = Autophagy-related 7, ARHI = aplasia Ras homolog member I.

A schematic illustration of intracellular mechanisms inducing cellular dormancy is presented in Figure 1.

### 4.2. Extracellular Mechanisms

Tumors comprise a complex mass of malignant, and non-malignant cells, along with extracellular matrix, the latter two collectively referred to as tumor microenvironment (TME). Crosstalk between components of TME and cancer cells either in primary or secondary sites plays a crucial role in initiating and maintaining tumor dormancy [6,61].

#### 4.2.1. Hypoxia

Among extracellular mechanisms, hypoxia has been seminally suggested as a fundamental contributor to tumor dormancy [62,63,64]. In their pioneer work, Holmgren et al. observed that while tumor cell proliferation did not significantly differ between cells in dormant masses and growing metastases, cells in dormant metastases exhibited a more than threefold increase in apoptosis rate. In the same study, it was shown that increased circulating levels of angiostatin were associated with tumor dormancy, suggesting that angiogenesis inhibitors might control metastatic growth by indirectly increasing apoptosis in tumor cells [65]. Other molecules expressed by endothelial cells in the microvasculature, which might influence tumor angiogenesis and therefore regulate the maintenance of tumor dormancy, include thrombospondin (TSP), vascular endothelial growth factor (VEGF), and epoxyeicosatrienoic acids (EETs) [66,67,68].

#### 4.2.2. Immune System

The immune system plays a key role in the maintenance of tumor dormancy [5,20]. An indirect indication of its role is the increased density of immune cells that have been observed in the bone marrow of patients with breast cancer displaying dormant DTCs [69]. In a lymphoma model, it was demonstrated that CD8+ cell depletion lifts cancer cell dormancy [70]. Similarly, in a melanoma model, dormancy was mediated, at least in part, by cytostatic CD8+ T cells, since depletion of these cells resulted in faster outgrowth of visceral metastases [71]. Natural killer (NK) cells, through perforin-mediated direct cytotoxicity, were shown to prevent metastases by reducing the number of circulating tumor cells in a mouse model of colon cancer. In the same study, mathematical processing of the data revealed that NK cells induced dormancy of malignant cells [72]. Moreover, in experimental models, metastasis-associated macrophages, which originate from inflammatory monocytes, were shown to release a specific cytokine that promoted metastatic seeding of breast cancer cells [73] and was associated with the production of an adhesion molecule mediating adhesion to lung tissue [74]. A lot of interest has been brought to cytokines and their effect on cancer cell dormancy. In an experiment involving breast cancer cells, the dormant cells with resistance to antiestrogen therapy highly expressed an interleukin-binding receptor, which was also predictive of treatment failure [75].

Additionally, immune evasion might be involved in cellular dormancy. It has been shown that dormant leukemia cells express programmed death-ligand 1 (PD-L1), allowing them to inhibit T-cells [61,76]. The microenvironment of quiescent DTCs may also contribute to immune evasion [61]. DTCs may become dormant by entering stem cell niches, which have been shown to be immune-protected sites. The hematopoietic stem cell niche, for example, is rich in CD4+CD25+ regulatory T-cells [77]. In a recent study, it was shown that an immunosuppressive T regulatory cell subpopulation inside the bone marrow (BM) niche promoted cancer cell quiescence through adenosine-mediated protection from oxidative stress and possibly immune killing [78]. Finally, the perivascular niche contributes to the suppression of immune responses through the expression of checkpoint ligands and secretion of immunosuppressive cytokines (for example, interleukin (IL)-6) [79].

#### 4.2.3. Stromal Cells and Extracellular Matrix

Additionally, stromal cells and associated factors might provide mechanical, physical, and chemical cues to disseminated tumor cells [80]. According to a recent study, the assembly of fibronectin matrix under transforming growth factor-β2 (TGF-β2) stimulation increased cancer cell survival by maintaining a dormant phenotype [81]. It has been shown that the abundance of TGF-β2 in TME is linked with the presence of dormant cells [82]. In an experimental model of HNSCC disseminated tumor cells, it was shown that TGF-β2 and TGF-β-RIII signaling through p38α/β regulated DTC dormancy and determined restrictive (bone marrow) or permissive (lung) microenvironments, therefore suggesting a “seed and soil” mechanism [82].

#### 4.2.4. Therapy-Induce Dormancy

Finally, anticancer therapy might represent another factor that promotes the acquisition of the dormant phenotype. Acquired resistance to anticancer treatment is commonly explained through the prism of the Darwinian theory, as the selection of best-adapted cells due to acquired genetic alterations; dynamic non-genetic heterogeneity of clonal cell populations may also exist and produce metastable phenotypic variants [83]. In response to cytotoxic therapy, the tumor cells might switch to a non-proliferative state. Interestingly, it has been suggested that the dose and duration of treatment may determine whether tumor cells become dormant or senescent, or trigger pro-apoptotic pathways [83]. Similar to quiescence, senescence is also characterized by growth arrest, however, it is considered to be an irreversible cell state [9]. Intriguingly, it has been shown that a subpopulation of cells undergoing therapy-induced senescence might acquire self-renewing properties and represent an avenue of dormant cells, with the potential to proliferate again [84,85]. Anticancer therapy may also induce dormancy indirectly, by creating an environment with external stress factors, such as hypoxia, nutrient deficiencies, and ROS generation that directly affect tumor cells [86,87].

## 5. Mechanisms of Escape from Dormancy

Cancer cells that originate from a primary tumor mass can disseminate to other tissues, where they might remain dormant and clinically undetectable for many years. Indeed, when residing at a distant site, DTCs confront a new, usually hostile microenvironment resulting in senescence or entry into dormancy [1,88]. In response to signals not yet fully elucidated, a small percentage of disseminated dormant cells reawaken, acquire metastatic potential, and form growing masses [6,9,88,89,90].

It has been suggested that alterations of TME might trigger the escape of DTCs from dormancy [91,92]. The deposition of type 1 collagen in the metastatic niche appears to affect the cytoskeletal organization and drives dormant breast cancer cells to proliferate through beta1-integrin signaling [93]. Moreover, it has been shown that thrombospondin-1 secreted by endothelial cells of a stable microvasculature favors quiescence. However, in the case of neovasculature, the secretion of TSP-1 not only promoted proliferation of cancer cells, but also growth rates were significantly accelerated [66]. Paradoxically, regarding microvascular homeostasis, it has been suggested that primary tumor removal might reawaken micrometastasis [94]. This could be explained by the loss of angiogenesis inhibitors such as angiostatin and endostatin secreted by the primary tumor, which is associated with inhibition of micrometastatic growth at distant sites [94,95].

Interestingly, chronic inflammation of the host tissue has been suggested as a trigger mechanism leading to escape from dormancy. In their recent work, Albrengues et al. showed that chronic inflammation of the lung caused by bacterial-derived lipopolysaccharide (LPS), or cigarette smoke can activate neutrophils to form neutrophil extracellular traps (NETs) that physiologically capture microorganisms. Further mechanistic analysis showed that NETs caused degranulation of the neutrophils and subsequent secretion of proteases such as neutrophil elastase (NE) and matrix metalloprotease 9 (MMP9) enhancing the processing of basement membrane laminin-111, which links to a3b1 integrin, resulting in the proliferation of dormant disseminated cells [96]. Another putative mechanism is that extracellular vesicles might be involved in the interaction between stromal cells and cancer cells leading to reawakening from dormancy [97]. In the liver, hepatic stellate cells have been shown to secrete soluble factors, including interleukin-8 (IL-8) and monocyte chemoattractant protein-1 (MCP-1) which promote the proliferation of breast cancer cells even under serum-starvation conditions possibly via ERK pathway activation [98]. Finally, age-associated changes in the bone secretome, involving upregulation of inflammatory cytokines, were associated with the proliferation of DTCs in the bone marrow microenvironment of aged mice [99].

Interestingly, the reawakening of dormant cells might be mediated by context-specific mechanisms in different metastatic niches as disseminated cells confront different challenges in each microenvironment. For example, in the bone marrow, a particularly vascularized area, the DTC may reside and stay dormant for prolonged periods [100]. Osteoclasts seem to play a crucial role in inducing escape from dormancy [101]. It has been shown that vascular cell adhesion molecule-1 (VCAM-1) recruits and activates osteoclasts resulting in a vicious cycle of bone destruction and tumor expansion [102]. Moreover, stromal injury in bone marrow can reawaken dormant breast cancer cells via the secretion of inflammatory cytokines IL-6 and IL-8 and activate TGFβ1 signaling [103]. In the case of the lung, another frequent metastatic site, several mechanisms have been implicated; Tank-binding kinase-1 (TBK1)-dependent promotion of proliferation of dormant breast cancer cells [104] and upregulation of periostin (POSTN) expression, leading to Wnt signaling [105]. Interestingly, Coco, an antagonist of TGF-β ligands, reactivated breast cancer cells in lung metastatic sites, but it was not associated with the promotion of bone or brain metastases [106], suggesting distinct implicated mechanisms in different target organs.

**Figure 2:**
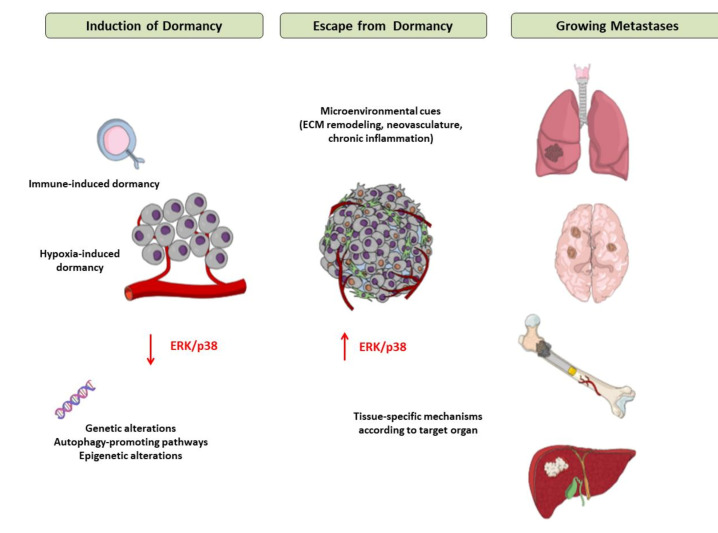
Mechanisms of induction of and escape from tumor dormancy. Tumor cells, when confronting a hostile environment, may enter a dormant state due to the interactions of extracellular factors (mainly hypoxia and immune cytotoxicity) and intracellular pathways. In response to signals that are not fully elucidated yet but appear to involve modifications of tumor microenvironment, such as extracellular matrix remodeling, neovascularization, chronic inflammation and tissue-specific mechanisms in each metastatic site, tumor cells escape from dormancy, start to proliferate and ultimately form macroscopic metastases in target organs. Abbreviations: ECM = Extracellular Matrix, ERK = Extracellular signalregulated kinase.

The main mechanisms of induction of and escape from tumor dormancy are illustrated in Figure 2.

## 6. Therapeutic Implications of Tumor Dormancy

Tumor dormancy, being the conceptual framework that explains the tumor latency period, provides an attractive opportunity for therapeutic interventions, aiming at minimizing the chances of relapse. Moreover, dormant tumor cells exist alongside fast-proliferating cells, even in periods of cancer progression, potentially representing a source of therapeutic resistance. Deciphering the mechanisms that regulate the conversion between dormancy and proliferation could direct novel therapeutic approaches to eliminate tumor cells [6]. However, among several implicated mechanisms, not all are relevant for clinical translation, and one must focus on the mechanisms that have more chances to succeed, for instance, those characteristics that distinguish dormant cells from other non-proliferating cells [31]. Although it is clear that dormant cells are the predominant origin of disease relapse in many tumor types, it remains elusive whether, when, and how the dormant cells should be treated [7,31,107,108]. Suggested therapeutic strategies regarding the dormant tumor cells are summarized in Table 1.

The main therapeutic approach aims to maintain residual cells in a permanent dormant state [7]. Hormone therapy represents the most successful example of this scenario since it directs cancer cells to enter the G0/G1 phase leading to proliferation arrest; it should be noted though that hormone therapy is not solely cytostatic [31]. Cell-cycle inhibition with the use of CDK inhibitors is considered to act similarly [109]. Several other agents have also been tested in preclinical models including thrombospondin 1 (TSP1), a glycoprotein produced from endothelial cells that has been shown to reduce the proliferation of invasive ductal carcinoma cells [110], inhibition of urokinase-type plasminogen activator receptor (uPAR) signaling [111], or the activities of ERK and Src kinases [112]. Given that this therapeutic strategy does not kill cancer cells, but rather keeps them inactive, long-term therapy is required, resulting in increased toxicity and allowing for cancer cells to acquire resistance [31,116].

Two other therapeutic approaches targeting tumor dormancy have been suggested. The first involves targeting cells that have acquired a dormant phenotype directly, and the second, and more controversial, proposes reawakening dormant cells [7]. Regarding the first strategy, the main challenge consists of the non-responsiveness of dormant cells to the majority of conventional cytotoxic therapies which target actively proliferating cells, thereby, different targets should be investigated [117]. For instance, inhibition of autophagy pathways in breast cancer cells was associated with reduced survival of dormant cells, but, not of cells that had switched to a proliferative state [43], implying that this strategy could be used in tumor latency periods. Epigenetic therapies are also being explored for the treatment of dormant cells via inhibition of epigenetic enzymes [113]. Regarding the second approach, the rationale behind reawakening dormant cells is that re-entrance to the G2/M phase will sensitize cells to cytotoxic therapy [7]. For instance, escape from quiescence via inhibition of APC(CDH1)-SKP2-p27(Kip1) signaling axis, enhanced the effect of imatinib in a preclinical model of gastrointestinal stromal tumors [114]. In a leukemia model, neutralization of osteopontin, which allowed dormant cells to start proliferating, acted synergistically with cytarabine to kill neoplastic cells [115]. Targeting the dormant cell microenvironment, which functions as a protective niche for dormant cells might re-sensitize the dormant cells to chemotherapy. The endosteal niche for example maintains dormancy by contact-dependent interactions with the tyrosine kinase AXL that is overexpressed by dormant myeloma cells. In a preclinical study, it was shown that the inhibition of AXL induced reawaking of dormant cells, sensitizing them to chemotherapy [118]. Another preclinical study reported that the use of integrin inhibitors led to a blockade of the dormant cell–vascular cell interaction and increased sensitivity for chemotherapy [119]. However, the major challenge of this treatment strategy is the possibility to unleash tumor cell proliferation promoting aggressive tumor behavior [7].

It is very important to note that several problems regarding the transfer of preclinical data to clinical trials exist. For example, evidence from preclinical studies has suggested RANKL (TNFSF11) as a target with strong potential to prevent breast cancer bone metastasis [120]. A phase 3 study (D-CARE) was conducted that combined a human blocking monoclonal antibody against the receptor for RANKL (denosumab) with standard-of-care adjuvant or neoadjuvant systemic therapy and locoregional treatments; nevertheless, it did not improve disease-related outcomes for women with high-risk early breast cancer [121]. The reasons for this outcome illustrate some of the problems regarding the clinical transfer of preclinical evidence of dormancy; it is possible that dosing schedules left a window for dormant DTC populations to become reactivated; it is also possible that chemotherapy causes severe alterations of the BM microenvironment which actually promotes the reactivation of dormant DTCs [9].

A limited number of therapeutic approaches regarding tumor dormancy are currently explored in clinical trials [7]. Interestingly, a phase 2 clinical trial is being conducted using hydroxychloroquine, an autophagy inhibitor, and/or mTOR inhibitors to treat patients with breast cancer who have completed primary therapy for breast cancer and harbor bone marrow disseminated tumor cells (clinical trial identifier NCT03032406) [7]. Another clinical trial (clinical trial identifier NCT03572387) recruits patients with disseminated prostate cancer that has not yet become detectable by imaging (PSA-only recurrence). This study uses 5-azacytidine and all-*trans* retinoic acid, which induces dormancy, to treat patients with prostate cancer after hormonal ablation [7,9].

## 7. Conclusions and Future Directions

Tumor dormancy represents an important step in cancer development, providing critical a window for therapeutic interventions. Unraveling the mechanisms of how tumor cells are kept dormant, and what reawakens them, are fundamental questions in cancer biology [9]. It seems that a plethora of intracellular pathways and extracellular factors are involved in the process, rewiring the cell to plastically alternate its metabolic and proliferative status. The phenomenon is highly dynamic, as it unfolds over time and space and it is regulated by the cooperation of cellular pathways and differential components of TME in different metastatic sites. Therefore, it is highly unlikely that the answers will be simple. However, mechanistic insights into cellular and tumor dormancy have provided the rationale for targeting this otherwise stable period of cancer development, in order to prevent recurrence and maximize therapeutic benefit [7]. It should be noted that the clinical translation of this research field is still in early steps, but it will likely expand in the near future.

It is important to underline that earlier research on this topic has been restricted by limitations regarding the characterization and identification of dormant cells on the one hand, and the reliable simulation of tumor microenvironment in experimental models on the other. Although there is a lack of specific biomarkers that could label dormant cells, major advancements in molecular biology and biotechnology have permitted the identification of dormancy-related signatures for certain types of cancer [45,122], as well as the development of sophisticated methods to accurately capture the cell cycle phase. However, an established characterization of the dormant phenotype is yet to be proposed. Additionally, experimental approaches to study tumor dormancy generally include in vitro, ex vivo, and in vivo models, all of which encounter challenges in recapitulating the complex dynamics of tumor niches in real-world patients [123]. The development of experimental models that could more accurately mirror the dynamics of tumor microenvironments, potentially with the use of advances in material bioengineering, in combination with mathematical and computational models to predict behavior in different contexts, will be key to objectively derive research results and transfer them to the real world. Finally, the development of methods to longitudinal monitor cellular and tumor dormancy in patients could facilitate clinical translation of research data by identifying the timing of clinical relevance of this biological process [9,123].

## Figures and Tables

**Table 1:** Suggested therapeutic strategies regarding tumor dormancy.

Treatment Strategy	Rationale	Disadvantages	Examples	Ref.
Maintaining dormant cells	To maintain tumor cells inactive and non-proliferating	Requires long-term therapy; toxicity; cost; acquired resistance	Hormone therapy, CDK inhibitors, Thrombospondin 1; urokinase-type plasminogen activator receptor (uPAR) signaling inhibition; ERK/Src inhibition	[109,110,111,112]
Targeting dormant cells	To eliminate tumor cells and minimize chances of relapse	Dormant cells are resistant to cytotoxic therapies; requires investigation of new targets	Inhibition of autophagy; Epigenetic therapies (for example inhibitors of histone demethylases)	[43,113]
Reawaking dormant cells	To reawake tumor cells to increase their susceptibility to anticancer therapy	May unleash uncontrolled proliferation; requires concomitant therapy with cytotoxic anticancer agents	Inhibition of APC(CDH1)-SKP2-p27(Kip1) signaling in combination with imatinib; neutralization of osteopontin in combination with cytarabine	[114,115]
